# Tandem mass tag-based proteomic profiling revealed potential therapeutic targets and mechanisms of liraglutide for the treatment of impaired glucose tolerance

**DOI:** 10.3389/fendo.2022.1031019

**Published:** 2022-11-14

**Authors:** Qiuyue Guo, Cong Han, Yunsheng Xu, Qingguang Chen, Xu Han, Sen Zhao, Jie Li, Hao Lu

**Affiliations:** ^1^ Diabetes Institute, Department of Endocrinology, Shuguang Hospital Affiliated to Shanghai University of Traditional Chinese Medicine, Shanghai, China; ^2^ Nephropathy Department, Affiliated Hospital of Shandong University of Traditional Chinese Medicine, Jinan, China; ^3^ Department of Endocrinology, Shandong Hospital of Integrated Traditional Chinese and Western Medicine, Jinan, China; ^4^ Medical School, Shandong University of Traditional Chinese Medicine, Jinan, China

**Keywords:** liraglutide (LIRA), impaired glucose tolerance (IGT), tandem mass tag (TMT) proteomics, protein targets, mechanism of action

## Abstract

**Objective:**

Based on the tandem mass tag (TMT) technique, our study investigated the potential therapeutic targets of Liraglutide (LIRA) on streptozotocin (STZ) induced impaired glucose tolerance (IGT) in rats and discuss the biological mechanism of the drug against IGT.

**Methods:**

10 rats were randomly selected from 31 male wistar rats of specific pathogen free (SPF) grade as control group and fed with conventional chow, offered the remaining rats a high fat and high sugar (HFSD) diet combined with an intraperitoneal injection of STZ to establish the IGT model, and excluded 2 non-model rats. Specifically, the model rats were randomly divided into Model group (n=10) and LIRA group (n=9). In addition, the LIRA group was subcutaneously injected with 0.06 mg/kg LIRA, during which the metabolic parameters including body weight and fasting blood glucose were recorded. After 8 weeks, samples were taken under anesthesia. Then, the cell morphology was observed using HE staining, and immunofluorescence was performed on the pancreatic tissues of the three groups of rats. Besides, the expression of differential proteins in pancreatic tissues of the three groups of rats was determined by the TMT proteomic labeling. Subsequently, Gene Ontology (GO) and Kyoto Encyclopedia of Genes and Genomes (KEGG) biological function analysis were performed on the intersection of Model and LIRA differential proteins.

**Results:**

LIRA could not only significantly reduce blood glucose levels but also improve islet cell morphology and function in IGT rats. Among the differential proteins between the model group and the blank group, 44 were reversed after LIRA treatment, of which 14 were up-regulated, while 30 were down-regulated, including PPIF, MPRIP, CYP51, TXNL1, BCL-2, etc. (FC>1.1 or<0.909, *P*<0.05). According to the GO and KEGG analysis results, it was related to biological processes such as fatty acid metabolism and adipocyte generation, which involved multiple signaling pathways regulating the function of islet cells, such as MAPK, PI, Ras, FcγR, and unsaturated fatty acids, and pyruvate metabolism.

**Conclusion:**

To sum up, LIRA participated in anti-IGT therapy through regulation of multiple target proteins and biological functions. This study is of great reference for further exploring the mechanism of action of LIRA at the protein level of IGT.

## 1. Introduction

Impaired glucose tolerance (IGT) refers to a special metabolic state, in which the fasting blood glucose (FBG) is below 7.0 mmol/L, and the 2h blood glucose after oral glucose tolerance test (OGTT) is between 7.8-11.0 mmol/L. Moreover, it is a necessary stage in the development of Type 2 Diabetes Mellitus (T2DM) as well as the important stage where T2DM can be controlled and reversed (1). At present, IGT becomes more common in young people. According to the IDF statistics, people suffering from impaired glucose tolerance are estimated to amount to 374 million in 2019 and may reach 454 million by 2030 and 548 million by 2045 (2). It is noteworthy that the etiology and pathogenesis of IGT have not been fully elucidated, and islet β-cell dysfunction and insulin resistance (IR) may be the main causes (3). Generally speaking, drugs including metformin and acarbose are mainly used to treat IGT. However, patients who take these two medicines tend to experience adverse reactions such as liver and kidney toxicity, stomach pain, and diarrhea (4). Therefore, it is of great significance to seek supplementary and alternative medicines to prevent and treat IGT.

It has been found that glucagon-like peptide-1 (GLP-1) can act on islet β-cells in a glucose concentration-dependent manner, stimulate insulin secretion, inhibit glucagon secretion, and reduce blood sugar (5). After endogenous GLP-1 is degraded by dipeptidyl peptidase-4 (DPP-IV), its biological activity is rapidly lost, and its half-life in blood is merely 1-2 min, so that it cannot be used to treat IGT (6). In fact, 97% of the amino acid sequence of LIRA, a novel long-acting GLP-1 receptor agonist, is overlapped with endogenous GLP-1. LIRA replaces one amino acid in the molecular structure of endogenous GLP-1 and adds a 16-carbon fatty acid chain, which prevents LIRA from being degraded and extends its half-life to 13h (7). As revealed by some studies, LIRA could lower blood sugar levels, improve islet cell function, and decrease the risk of adverse cardiovascular events (8). Besides, myocardial ischemic contracture was reduced in myocardial ischemic-reperfused injury rats in the IGT + LIRA group (9). In comparison to the Model, IGT mice after LIRA intervention had higher C-peptide levels after the OGTT test, indicating that LIRA can improve islet β-cell function in IGT rats (10). In addition, serum Fetuin-B expression was significantly decreased in IGT patients after LIRA intervention (11). At present, the precise target of LIRA in the treatment of IGT remains unclear. Our novelty lies in that we pay attention to the IGT through TMT technology, with a view to focus on the early development of T2DM. Proteomics can identify and analyze changes in proteins at the overall cellular level. By detecting abnormal changes in protein expression, it can not only clarify the occurrence and development of diseases and potential targets of drugs, but also provide comprehensive information on cell dynamics (12). As a high-throughput screening technology, TMT technology uses multiple isotopic reagents to label the N-terminal or lysine side chain group of protein polypeptides and conducts high-precision tandem mass spectrometry, which has been commonly applied in quantitative proteomics in recent years (13).

Through analyzing TMT marker proteomics, this study constructed an IGT rat model to explore the potential protein targets of LIRA in the treatment of IGT as well as its possible biological pathways, providing new research ideas and directions for the prevention and treatment of IGT and T2DM.

## 2 Methods and materials

### 2.1 Laboratory animals

In this research, 31 five-week-old male wistar rats of SPF grade weighing (130 ± 10)g were purchased from Jinan Pengyue Biological Breeding Co., Ltd. (#1107261911005606), and then raised in the Experimental Animal Center of Shandong University of Traditional Chinese Medicine (Temperature: 18-22°C, humidity: 60%-70%, light-dark cycle: 12h, 5 animals/cage, SPF level feeding, free food and water). During the experiment, all the procedures were strictly implemented in accordance with Chinese guidelines, including *Laboratory Animal - Requirements of Environment and Housing Facilities* (GB14925-2001) and *Guidelines for Humane Handling of Laboratory Animals* (MOST 2006a). Apart from that, all the animal experiments were approved by the Animal Ethics Committee of Shandong University of Traditional Chinese Medicine (No. SDUTCM20190520001).

### 2.2 Model preparation and grouping

**Figure 1:**
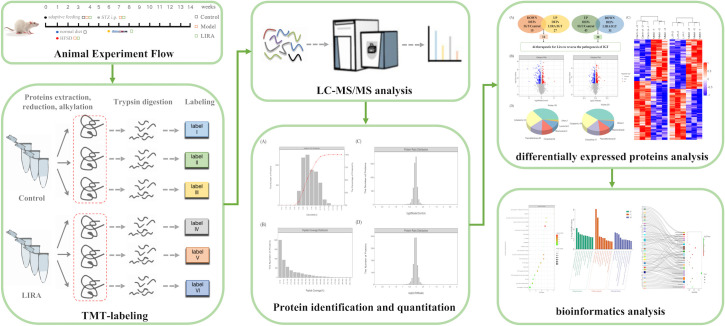
Flow chart of animal experiments. The diagram of TMT marker quantitative proteomics analysis operation mainly included steps such as protein extraction, peptide enzymatic hydrolysis, TMT labeling, chromatographic fractionation, liquid chromatography-tandem mass spectrometry (LC-MS/MS) data collection, data retrieval, GO and KEGG biological enrichment functional analysis, etc. LIRA, Liraglutide; STZ, StrepStozocin; ip, intraperitoneal injection; HFSD, high-fat and high-sugar diet.

After 31 male Wistar rats of SPF grade were adaptively fed for 1 week, 10 rats were randomly selected as Control group, and the rest were fed with HFSD (19093212, Keao Xieli Feed Co., Ltd., Beijing), which were composed of 67% conventional feed, 10.0% lard, 20.0% sucrose, 2.5% sodium cholate, and 2.5% cholesterol. Referring to the previous studies and related literature, rats in the model group were given a one-time intraperitoneal injection of STZ 15 mg/kg (18883-66-4, Sigma, United States). STZ was dissolved in 0.1 mmol/L sodium citrate (2018010203, Zhiyuan Company, Tianjin) and citric acid (2018-09-16, Dingshengxin Company, Tianjin) buffer, pH=4.4, final concentration was 4%, used right after it was ready, and used up within 20min), whereas the rats of Control group were injected intraperitoneally with citrate buffer (pH=4.4) at a dose of 0.1ml/L. In the wake of 72 hours, blood was collected from the tail vein of the rats, and the FPG and 2hPG values of the rats were measured by OGTT test. If FPG of three OGTT tests performed on different days were all <7.0mmol/L and 7.8mmol/L ≤ 2hPG <11.1mmol/L, it could be considered that the modeling was successful. What’s more, two non-model rats were excluded, and the remaining model rats were randomly divided into Model group (n=10) and LIRA group (n=9). More specifically, LIRA rats were subcutaneously injected with 0.06 mg/kg LIRA (J20180026, Novo Nordisk, Denmark) (14), whereas Control and Model rats were given equal volumes of normal saline. In addition, the body weight and OGTT test results (portable blood glucose meter: Johnson & Johnson, USA) of each group were measured at a fixed time every week. For the rats in each group, they were allowed to drink water freely and fasted for 12 hours after administration for 8 weeks (**Figure 1**).

### 2.3 HE staining to observe the morphology and structure of islet cells in three groups of rats

An appropriate amount of rat pancreatic tissue was fixed in 10% formaldehyde solution for 12 h, dehydrated in ethanol, embedded in paraffin (after immersed in xylene), cut into slices with a thickness of 4 μm, dried at 45°C, and then HE stained and mounted. Afterwards, the light microscope was used to observe the morphology and distribution of islet cells.

### 2.4 Immunofluorescence detection of the localization and distribution of islet α/β cells

Paraffin sections (LEICA company, Germany) were deparaffinized and rehydrated in graded alcohols. In addition, antigens were retrieved, autofluorescence quencher was added, and bovine serum albumin was added for incubation. Furthermore, primary antibodies anti-insulin mouse mAb (GB13121, Servicebio, China) and anti-glucagon rabbit pAb (GB13097, Servicebio, China) were added dropwise to the sections, incubated overnight, washed, incubated with corresponding secondary antibodies, and washed. Besides, DAPI (Solarbio, C0060) staining solution was added dropwise, and anti-fade mounting medium was adopted to mount the slides. Then, images were observed and collected under the fluorescence microscope (Leica DM2500 microscope: Leica, Germany).

### 2.5 Quantitative proteomic analysis of TMT markers

This study utilized TMT quantitative proteomics technology to analyze the expression of differential proteins in the three groups (**Figure 1**), randomly selected three pancreatic samples from each group, extracted the protein by SDT lysis buffer (4% (w/v) SDS, 100mM Tris/HCl pH7.6, 0.1M DTT), and quantified the BCA. What’s more, filter-aided proteome preparation (FASP) was used for trypsin digestion, followed by peptide quantification (OD280). Afterwards, the peptides were labeled according to the instructions of the TMT labeling kit (Thermo Company) and fractionated using the High pH Reversed-Phase Peptide Fractionation Kit. Beyond that, each sample was separated using the HPLC liquid phase system Easy nLC (Thermo Scientific, USA), and then mass spectrometry analysis was made using a Q-Exactive mass spectrometer (Thermo Scientific, USA). Furthermore, data were identified and quantified using software Mascot 2.2 and Proteome Discoverer 1.4. With fold change (FC)>1.1 folds (up-regulation>1.1-fold or down-regulation<0.909-fold) and <0.05 as the standard, the number of differentially expressed up-regulated and down-regulated proteins between groups was screened out for cluster analysis. Based on the R package, two dimensions of sample and protein expression were classified, and a hierarchical clustering heat map was generated. Apart from that, CELLO was used to perform Subcellular localization prediction. Notably, the intersection of the up-regulated differentially expressed protein targets (Model vs Control) and the down-regulated differentially expressed protein targets (LIRA vs Model) is the specific target that is up-regulated after the onset of IGT and improved after LIRA treatment. At the same time, the intersection of the down-regulated differentially expressed protein targets (Model vs Control) and the up-regulated targets (LIRA vs Model) is the specific target that is down-regulated after the onset of IGT and improved after the LIRA treatment. In order to further explore the functions of the proteins in cells, the subcellular localization analysis of all differentially expressed proteins was performed using the subcellular structure prediction software CELLO. Beyond that, GO and KEGG functional annotations were performed on the differential proteins between LIRA and Model to clarify the biological processes and signaling pathways involved in LIRA treatment of IGT. Moreover, Blast2 GO was used to perform GO annotation analysis on the target protein set, and KAAS software was adopted to conduct KEGG pathway annotation on the target protein set.

### 2.6 Statistical analysis

All the data were statistically analyzed by SPSS 24.0 software and the experimental data were expressed by `X ± S (mean ± sd). In addition, the SNK test was used for the comparison between groups, the paired t-test was performed for the comparison within groups, and the chi-square test (χ^2^) was applied to analyze count data. P*<*0.05 was considered statistically significant.

## 3 Results

### 3.1 Comparison of body weight, FBG, and 2hPG among the three groups of rats (n=9)

**Figure 2:**
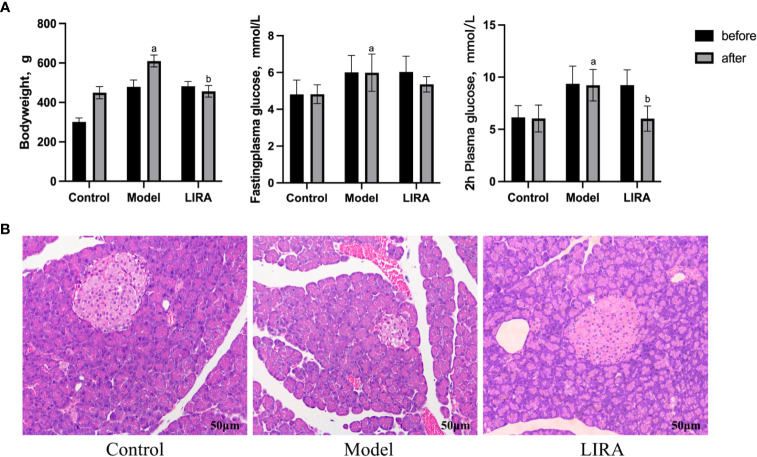
**(A)** Changed of body weight/FPG/2hPG of rats in each group before and after intervention (n=9). Compared with Control group, ^a^ *P*<0.05; Compared with Model group, ^b^ *P*<0.05. **(B)** The morphology and structure of islets were observed by HE staining (200×). From left to right are control, model and LIRA (n=3).

After 8 weeks of intervention, the body weight of the Model rats increased in comparison to that of the Control rats (*P<*0.05), whereas the body weight of LIRA rats was lower than that of Model rats (*P<*0.05), indicating that the body weight of IGT rats could be reduced by LIRA. Compared with Control rats, the FPG of Model rats increased (*P<*0.05). In comparison to the Model rats, FPG of LIRA rats decreased, but the difference was not statistically significant (*P*>0.05). After treatment, in comparison to the Control rats, 2hPG of Model rats increased (*P<*0.05), and 2hPG decreased after LIRA intervention (*P<*0.05), indicating that LIRA could reduce 2hPG in IGT rats (**Figure 2A**).

### 3.2 HE staining to observe the morphology and structure of rat pancreas (n=3)

The islet tissue of the Control rats had clear borders, with the surrounding tissue being evenly distributed. Besides, the islet cells were arranged regularly, with uniform size and a large number. In comparison to the pancreatic tissue of the Control rats, the pancreatic tissue of the IGT rats which were established by HFSD combined with STZ had unclear borders. Meanwhile, the islet cells were disordered, irregular in size and shape, and few in number. In comparison to IGT rats, the islets of LIRA rats had clear borders and regular round shapes. Besides, the islet cells were arranged relatively neatly, and their number increased relatively (**Figure 2B**).

### 3.3 Immunofluorescence observation of islet cells (n=3)

**Figure 3:**
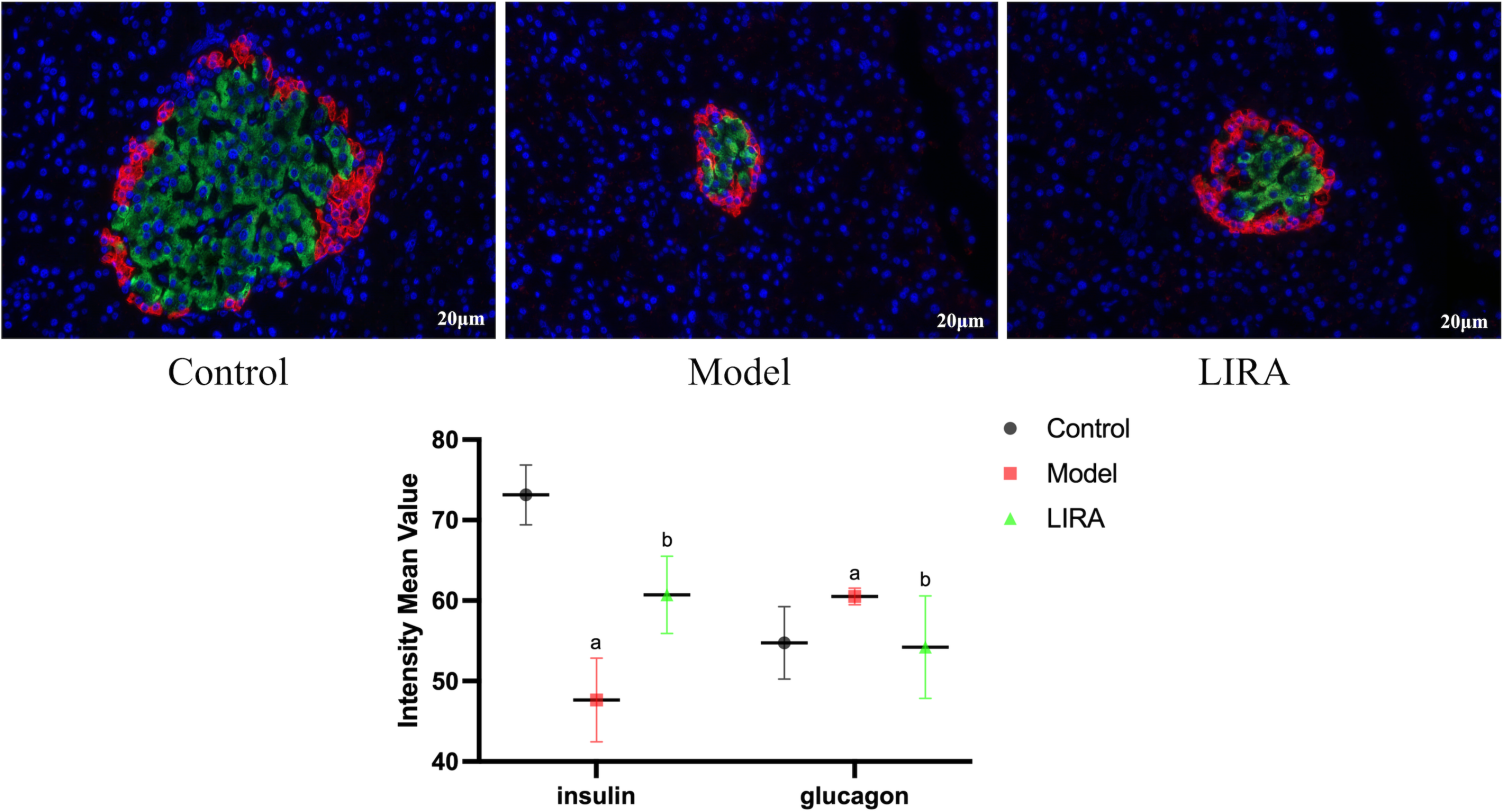
Immunofluorescence observation of islet α/β cells (400X light microscope). The red fluorescence were insulin staining, representing α cells; the green fluoresces were glucagon staining, representing β cells (n=3). Compared with Control group, ^a^ *P*<0.05; Compared with Model group, ^b^ *P*<0.05.

The islet β cells of the Control rats were aggregated in the center of islet, with a large number and uniform distribution, islet α cells were distributed around β cells, with a few numbers and uniform distribution. In comparison to the Control group, the number of islet β cells in the Model rats decreased, and the distribution of islet cells were uneven, the number of islet α cells increased, the distribution of the islet α cells was uneven. Compared to Model group, the number of islet β cells in LIRA rats increased and the islet β cells aggregated in the center of islet, the islet α cells, which were small in number, were mainly distributed in the periphery of the islet (**Figure 3**).

### 3.4 Analysis of TMT proteomics results (n=3)

#### 3.4.1 Quality control analysis

**Figure 4:**
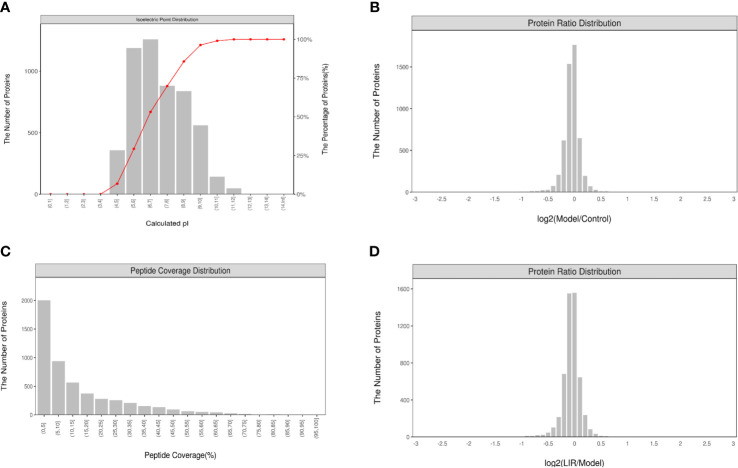
Quality control analysis and characterization of peptides (n=3). **(A)** Peptides identified distribution; **(B)** Protein sequence coverage distribution; **(C)** Distribution of protein abundance ratios between Model and Control. **(D)** Distribution of protein abundance ratios between LIRA and Model. The data showed that the quality control analysis and the experimental data were reliable.

In this experiment, the quality deviations of all identified peptides were mainly distributed within 10 ppm, indicating that the quality deviations were small, and the identification results were accurate and reliable (**Figure 4A**). As shown by the MASCOT analysis, MS2 had an ideal MASCOT score, in which more than 51.31% of the peptides scored more than 20 points, and the median peptide scored 21 points, revealing that the quality of MS experimental data was credible (**Figure 4B**). What’s more, the abundance ratio of most proteins approached 1 in the two groups of equally labeled samples (**Figures 4C, D**). According to the above results, this experiment has maintained a good quality deviation throughout the whole process, and the experimental data collected are credible.

#### 3.4.2 Identification of differential protein DEPs between groups and potential protein targets of LIRA

**Figure 5:**
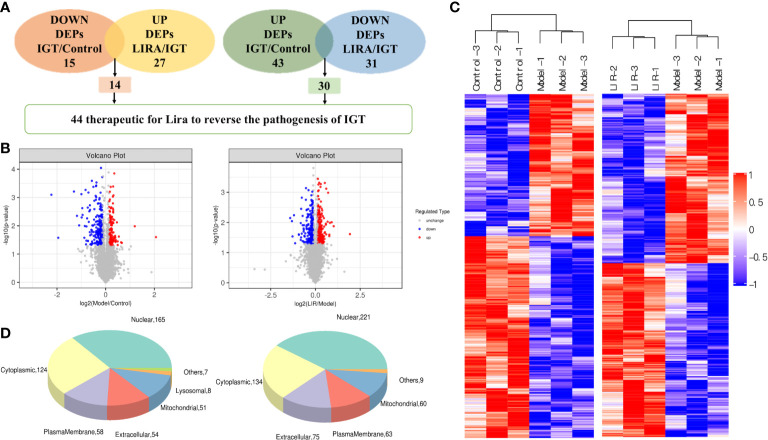
**(A)** The number of differentially expressed proteins among three groups (n=3). **(B)** Volcano plots of significantly differentially expressed protein targets among the three groups. dots in the volcano plot represent differential proteins, red represents up-regulation, green represents down-regulation, and black represents differential proteins that did not meet the screening conditions and were excluded. The left figure represents Model/Control differential protein screening, and the right figure represents LIRA/Model differential protein screening. **(C)** Heat map of significantly differentially expressed proteins between groups. the horizontal axis represents the sample number, the vertical axis represents the differential protein, the color represents the relative expression of the differential protein, and clustering analysis was performed on the differential proteins with similar expression. **(D)** The subcellular localization of differentially expressed proteins among the three groups. The left panel showed the subcellular localization of Model/Control differential proteins; the right panel showed the subcellular localization of LIRA/Model differential proteins.

According to the screening criteria (FC>1.1 or <0.909, *P*<0.05), the proteomic analysis of the three groups of samples revealed that 43 differential proteins of Model/Control were up-regulated, and 30 were down-regulated after drug intervention(**Figure 5A**). To be specific, the target proteins are PAPSS1, DAD1, CIAO1, DLGAP4, DCAF11, NSA2, ADAM10, PDCD10, PTPA, G-septin, Rmdn2, LRRC59, MRPS2, HMOX2, BET1L, ECI1, YIF1B, AGFG2, ATP5MF, SUB1, UGT2B10, ACOT2, BCL2, MEN1, PSMb6L, ATG2B, Serpina3c, Afm, LYRM4, and ENY2 respectively. In the wake of LIRA treatment, 15 differential proteins of Model/Control were down-regulated (**Table 1**) and 14 were reversed, namely TBC1D13, TCEAL9 (WBP5), PPIF, ME2, MKK6 (MAP-KK6), MPRIP (MRIP), SELENOF, CYP51, HDAC6, SLC6A9, DNASE2, SelT, FKBP8, TXNL1 (**Table 2**). Based on the comprehensive comparison, the potential protein targets of LIRA in IGT model rats were the above 44 differential proteins, suggesting that LIRA regulates the differential expression of these 44 proteins, so as to treat IGT model rats, regulate islet cell function, and lower blood sugar levels. Apart from that, volcano plots are drawn to clearly display significant differences in proteins between groups (**Figure 5B**), and clustering analysis was performed on target proteins in the form of Heat-map (**Figure 5C**).

**Table 1:** Down-regulated pancreatic DEPs in LIRA compared with Model rats (n=3).

Accession	Protein Name	Model/Control		LIRA/Model	
		FC	P. value	FC	P. value
ENSRNOP00000071489	PAPSS1	1.183	0.002	0.908	0.007
ENSRNOP00000012233	DAD1	1.203	0.017	0.907	0.030
ENSRNOP00000017603	CIAO1	1.184	0.004	0.906	0.011
ENSRNOP00000034166	DLGAP4	1.148	0.015	0.901	0.001
ENSRNOP00000074435	DCAF11	1.179	0.000	0.898	0.002
ENSRNOP00000022138	NSA2	1.184	0.029	0.897	0.034
ENSRNOP00000073306	ADAM10	1.299	0.001	0.895	0.022
ENSRNOP00000013585	PDCD10	1.111	0.043	0.890	0.027
ENSRNOP00000024914	PTPA	1.215	0.005	0.888	0.045
ENSRNOP00000010491	G-septin	1.203	0.014	0.887	0.045
ENSRNOP00000008045	Rmdn2	1.155	0.015	0.886	0.013
ENSRNOP00000004941	LRRC59	1.286	0.000	0.885	0.017
ENSRNOP00000013548	MRPS2	1.106	0.026	0.884	0.010
ENSRNOP00000075102	HMOX2	1.131	0.021	0.884	0.023
ENSRNOP00000017684	BET1L	1.119	0.019	0.871	0.024
ENSRNOP00000011784	ECI1	1.126	0.001	0.868	0.008
ENSRNOP00000053771	YIF1B	1.153	0.030	0.861	0.028
ENSRNOP00000074192	AGFG2	1.174	0.026	0.857	0.037
ENSRNOP00000029426	ATP5MF	1.115	0.005	0.856	0.034
ENSRNOP00000067467	SUB1	1.188	0.037	0.855	0.040
ENSRNOP00000069054	UGT2B10	1.204	0.014	0.843	0.018
ENSRNOP00000013515	ACOT2	1.298	0.005	0.832	0.012
ENSRNOP00000003768	BCL2	1.195	0.023	0.826	0.030
ENSRNOP00000028592	MEN1	1.182	0.002	0.812	0.033
ENSRNOP00000015747	PSMb6L	1.398	0.031	0.765	0.037
ENSRNOP00000006131	ATG2B	1.348	0.035	0.728	0.015
ENSRNOP00000013896	Serpina3c	1.248	0.029	0.726	0.034
ENSRNOP00000057275	Afm	1.184	0.030	0.720	0.009
ENSRNOP00000065815	LYRM4	1.364	0.010	0.717	0.020
ENSRNOP00000006402	ENY2	1.449	0.042	0.643	0.025

IGT, impaired glucose tolerance; LIRA, Liraglutide; FC, fold change.

**Table 2:** Up-regulated pancreatic DEPs in LIRA compared with Model rats (n=3).

Accession	Protein Name	Model/Control		LIRA/Model	
		FC	P. value	FC	P. value
ENSRNOP00000021431	TBC1D13	0.697	0.020	2.053	0.012
ENSRNOP00000050036	TCEAL9	0.462	0.006	1.772	0.007
ENSRNOP00000014382	PPIF	0.800	0.027	1.349	0.003
ENSRNOP00000073241	ME2	0.542	0.007	1.334	0.024
ENSRNOP00000006217	MKK6	0.710	0.035	1.306	0.039
ENSRNOP00000069198	MPRIP	0.792	0.026	1.284	0.013
ENSRNOP00000075579	SELENOF	0.715	0.006	1.281	0.010
ENSRNOP00000009985	CYP51	0.882	0.009	1.249	0.005
ENSRNOP00000063689	HDAC6	0.890	0.023	1.236	0.003
ENSRNOP00000070161	SLC6A9	0.863	0.045	1.231	0.000
ENSRNOP00000014887	DNASE2	0.899	0.050	1.192	0.028
ENSRNOP00000072567	SelT	0.883	0.034	1.157	0.020
ENSRNOP00000074539	FKBP8	0.836	0.002	1.146	0.000
ENSRNOP00000074049	TXNL1	0.873	0.012	1.113	0.027

### 3.5 Biological function analysis (n=3)

It can be observed from the data that most of the differential proteins between the two groups were located in the nucleus and cytoplasm (221, 134/165, 124), followed by extracellular matrix, plasma membrane and mitochondria (75, 63, 60/54, 58, 51), and some differential proteins were localized in other places (e.g., endoplasmic reticulum and Golgi). In addition, 3 of the differentially expressed proteins between LIRA/Model were located in lysosomes (**Figure 5D**).

**Figure 6:**
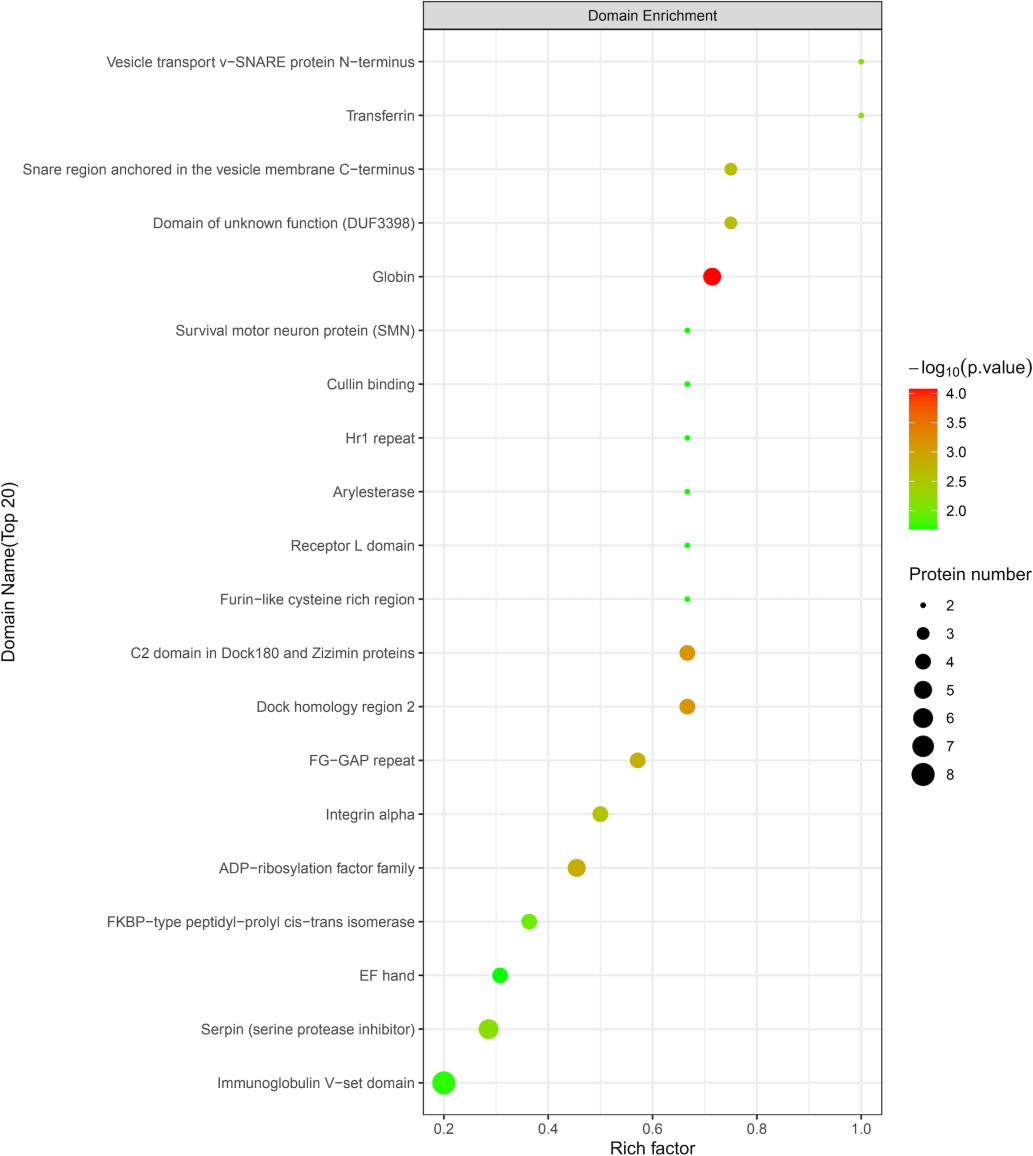
LIRA/Model differential protein domain analysis diagram (n=3). The horizontal axis is the rich factor (Rich factor ≤ 1), and the vertical axis represents the statistical results of DEPs under each domain classification. The color of the bubbles indicates the significance of enrichment under the corresponding domain classification, the P value is calculated by Fisher’s Exact Test, and the color gradient indicates -log10 (P value). The redder the color, the smaller the P-value, and the higher the significance level of the enrichment under the corresponding domain classification.

**Figure 7:**
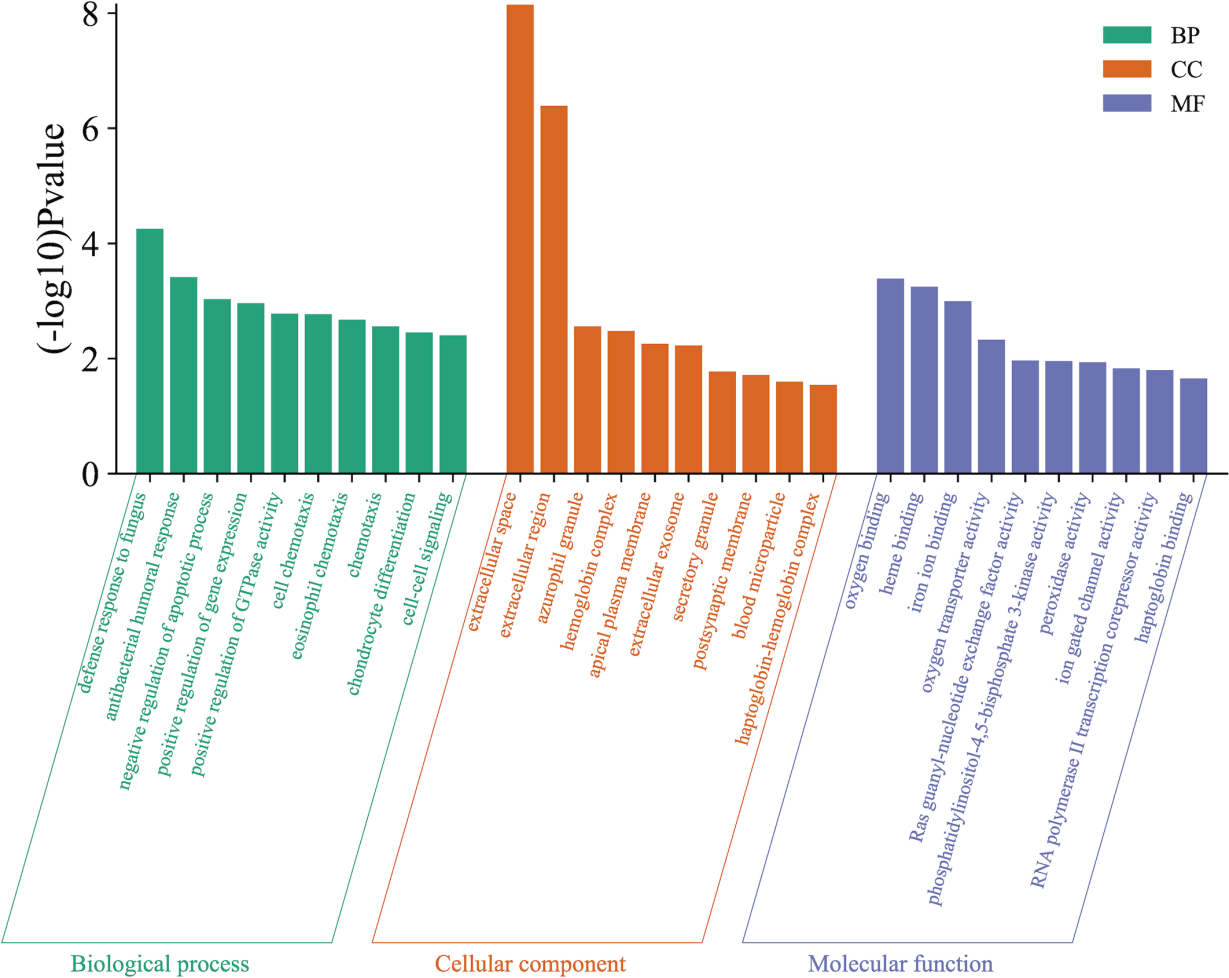
GO functional enrichment analysis (n=3). The figure shows the top 10 related enrichment pathways of each GO functional analysis.

**Figure 8:**
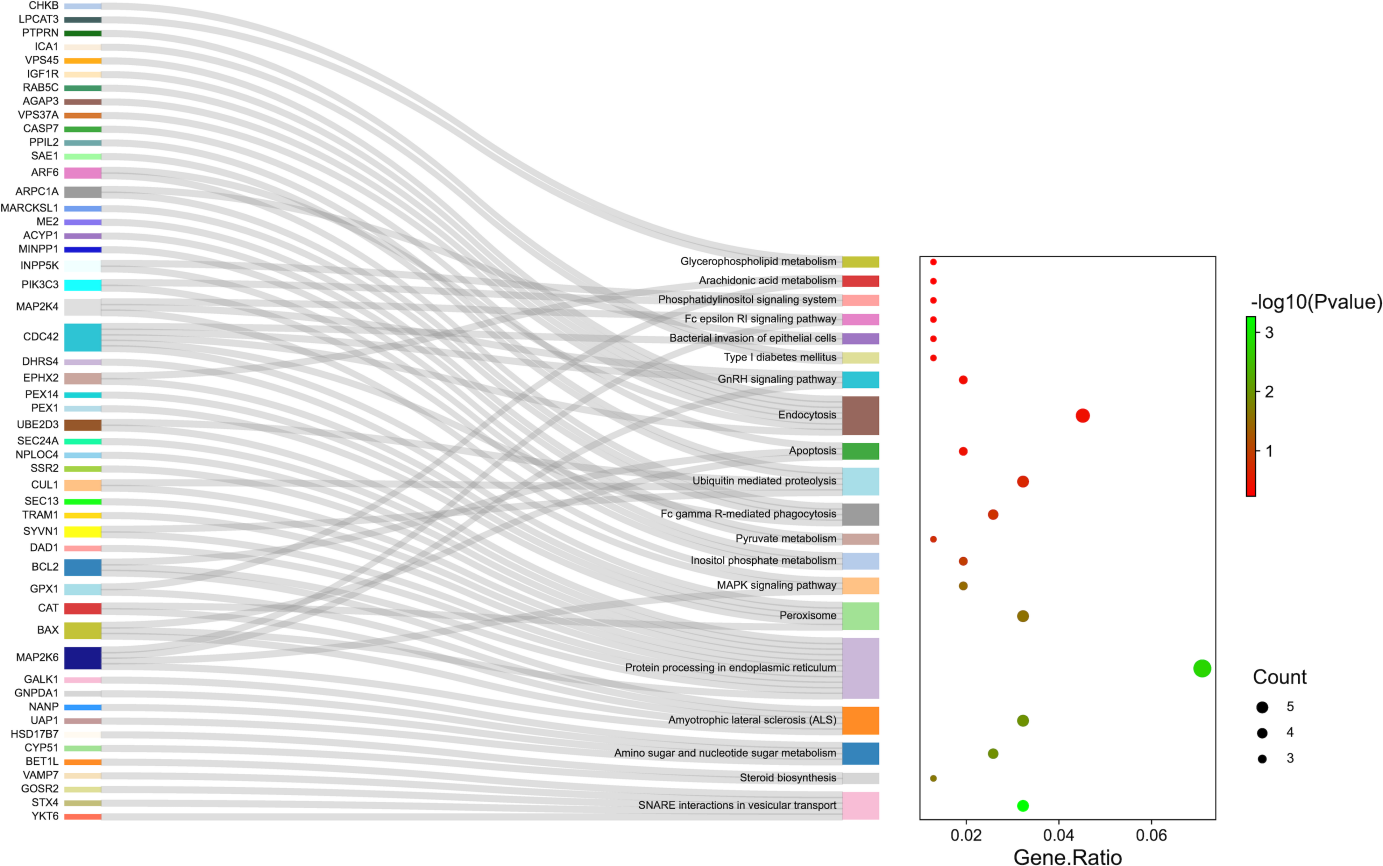
KEGG pathway enrichment analysis (n=3). The genes associated with the enrichment pathway are represented on the left. The bubble area size represented the number of genes belonging to the pathway in the target gene, and the bubble color represented the enrichment significance, i.e., the size of the P value. The top 10 pathways enriched by KEGG were shown in figure.

According to the protein domain analysis, the domain of DEPs was mainly enriched in the Vesicle transport -SNARE protein N-terminus, Transferrin, C2 domain in Dock180 and Zizimin proteins, and Globin, etc (**Figure 6**). Then, GO analysis indicated that the action pathways of LIRA in the treatment of IGT mainly include apoptosis regulation process (GO:0043066), gene expression regulation (GO:0010628), positive regulation of GTPase activity (GO:0043547), cell chemotaxis (GO:0060326), regulation of Ras active factor (GO:0005088), etc. (**Figure 7**). As suggested by the results of KEGG analysis, the anti-IGT signaling pathway of LIRA may not only be related to the activation of MAPK, FcϵRI, GnRH, P13K and other signaling pathways, but also be associated with FcγRI-mediated phagocytosis, apoptosis, ubiquitinated proteolysis, unsaturated fatty acid metabolism and aldosterone metabolism (**Figure 8**).

## 4 Discussion

IGT is a metabolic disease between normal glucose tolerance and type 2 diabetes mellitus, which may be accompanied by obesity, insulin resistance and other diseases. It is mainly manifested as impaired fasting blood glucose and postprandial blood glucose. In the case of normal glucose regulation, early-phase insulin secretion may become impaired, and further aggravation is very likely to progress to IGT. As IGT progresses to the course of T2DM, islet β-cell function will decrease progressively. Therefore, the key for treating T2DM is to conduct the early intervention in the IGT phase, so as to protect islet β-cells and alleviate β-cell hypofunction. LIRA is a long-acting GLP-1 receptor agonist with 97% homology to native GLP-1. As shown by evidence, LIRA has anti-islet β-cell apoptosis properties and can prolong islet β-cell lifespan in patients with type 2 diabetes mellitus (10). It has been found in the previous study that the miRNA expression profile of STZ-induced diabetic rat pancreatic tissue is abnormal. Beyond that, high-throughput sequencing could identify 9 target miRNAs for LIRA to treat diabetes, involving autophagy, Fox O, PI, HIF-1, and other signaling pathways (15). In this study, TMT proteomics technology was used to screen out 44 potential protein targets of LIRA in the treatment of IGT, including TBC1D13, PPIF, MPRIP, ME2, CYP51, DAD1, PTPA, TXNL1, ATG2B, BCL-2, etc., suggesting that these target proteins may be associated with insulin resistance, islet beta cell insufficiency, etc. At the same time, LIRA can change the expression of the above differential proteins, making them more likely to be in the blank group. That is to say, LIRA improves insulin resistance and islet function in IGT rats to a certain extent by affecting glucose transport, apoptosis, lipid metabolism, and autophagy, among other pathways.

According to the previous studies, the expression of the above differential proteins is related to glucose and lipid metabolism, insulin resistance, and insufficiency of islet cell function. For example, it has been confirmed that PPIF is related to FBG levels, insulin sensitivity and islet cell function in the diabetic rats. In the skeletal muscle of diabetic rats, Atp5, Ant1 and PPIF mRNA levels are decreased, while Ant2 mRNA levels are increased (16). In this experiment, it was found that the expression of PPIF in the model group was decreased, and the expression of PPIF was significantly increased after LIRA intervention (P=0.003), revealing that LIRA may reduce blood sugar levels by increasing insulin sensitivity and improving islet cell function in IGT rats. In a clinical study of more than 20,000 people, it had been proved that human vitamin E-binding glycoprotein Afamin was positively associated with HOMA-IR in type 2 diabetes mellitus (β=0.110 [95%CI 0.089-0.132], P=1.37×10^-23^) (17). In addition, some studies demonstrated that Menin, the protein encoded by MEN1, can interact with various epigenetic mediators to regulate gene transcription, and inhibit pancreatic β-cell proliferation. Meanwhile, Menin improves islet β-cell function and reduces blood sugar levels in diabetic rats (18). Besides, CYP51 has been confirmed to get involved in glucose and lipid metabolism, and astragali powder can significantly up-regulate the expression of this gene in obese rats induced by a high-fat diet, which is consistent with the results of this study (19). Moreover, there is a potential relationship between the nuclear protein Eny2 and insulin secretion (20). Inhibiting the expression of Eny2 induces an increase in the level of incretin. LIRA reduces the level of blood glucose in IGT rats by down-regulating the expression of Eny2 in our study. Therefore, we presumed that the mechanism of action of LIRA may be related to the regulation of the secretion of incretin. On the basis of previous studies, it can be observed the expression of ME2 was elevated in db/db rats, which was reversed after ATR treatment. This finding is consistent with the results of this study (21).

In addition to the above findings, the mechanism by which LIRA treats IGT and improves blood glucose levels in rats may also be related to the regulation of islet cell apoptosis, oxidative stress, and reduction of inflammatory responses. According to the experimental data, LIRA can up-regulate the expression of anti-apoptotic gene DAD1 (22), which was consistent with the results of our study. As pointed out by He C et al., in HFSD-induced diabetic rats, in comparison to the control group, the blood glucose level of rats was significantly decreased after the intervention of 10% Yunvjian medicated serum, and the mechanism may be down-regulation of autophagy protein ATG2B to protect INS-1 cells from glucolipid toxicity-induced apoptosis (FC=0.914). This finding is in line with the trend in this study (23). The data showed that in comparison to the control group, the expression of insulin and apoptosis protein Bcl-2 in the model group decreased, while the insulin level and the expression of Bcl-2 protein increased significantly after probiotic intervention (*P<*0.01). That is to say, LIRA might improve the survival rate of islet cells, inhibit cell apoptosis, and improve islet cell function by regulating the expression of Bcl-2 protein, so that the purpose of anti-IGT can be achieved (24). In fact, Selenoprotein T (SelT) is a redox protein. This study found that LIRA can reverse the expression of SelT in the model group, which may be related to oxidative stress, etc (25). TXNIP, which is involved in the activation of the NLRP3 inflammasome, is closely related to the onset of type 2 diabetes mellitus (26). As claimed by Bai S et al, MMP-2 is related to the progression of chronic diabetes complications, and exosomal circ_DLGAP4 regulates the expression of miR-143 and targets the ERBB3/NF-κB/MMP-2 axis, which triggers the occurrence of diabetic nephropathy (27). In addition, some studies have argued that MRIP is related to vasodilatory function. By combining with MYPI1, it activates the vasodilatory signaling pathway. At the same time, the expression of MRIP decreases in a high-glucose environment, the interaction with MYPI1 diminishes, and the vasodilation function declines, leading to hypertension in diabetic patients (28). In this study, the expression of MRIP in the model group was decreased, and the expression was up-regulated after LIRA intervention, indicating that LIRA can improve the vasodilation function of diabetic rats, and the mechanism may be related to the vasodilatory signaling pathway mediated by insulin resistance. As suggested by previous studies, the ADAM10 and ADAM17 downregulate STZ-induced type 1 diabetes mellitus in rats, and the mechanism is related to atherosclerosis (29).

According to the functional enrichment analysis, the LIRA reduces blood glucose levels in IGT rats and improves islet cell function, which involves multiple signaling pathways such as MAPK, PI, FcγR, FcϵRI, as well as unsaturated fatty acids and pyruvate metabolism, apoptosis, and endocytosis. The study found that the natural isoquinoline alkaloid palmatine (PAL) can help improve high-fat diet (HFD)-induced insulin resistance in IGT rats, increase islet β-cell proliferation, and strengthen islet cell function. The mechanism may activate MAPK signaling pathway through the regulation of JNK signal transduction, thereby improving insulin deficiency (30). FcγR and its ligands are closely related to the pathogenesis of obesity and T2DM. As shown by some studies, the FcγR not only blocks insulin-induced phosphorylation of AKT and FOXO1, but also up-regulates the expression of G6Pase and PEPCK mRNA in a high-glucose environment, which reveals the potential role of FcγR in regulating glycolipid metabolism (31). Beyond that, a Mendelian randomized trial found a positive correlation between type 2 diabetes mellitus and the prevalence of amyotrophic lateral sclerosis (ALS) in Asian populations. As shown by the Bioinformatic analysis of this study, the mechanism of action of LIRA in the treatment of IGT is associated with ALS-related pathways. LIRA may play a potential role in decreasing the prevalence of ALS in patients, and the possible mechanism needs to be further studied (32). In addition, KEGG analysis revealed that the anti-sugar mechanism of LIRA may also be related to the intestinal flora. As confirmed by some studies, LIRA regulates insulin secretion in obese rats by targeting the gut microbiota and gut immune system (33). According to the bacterial 16S rRNA sequencing analysis, among the patients who were treated with LIRA, the alpha diversity of gut microbes was reduced, the distribution of microbiota structure was altered, and the interactions of microbes were changed, suggesting that LIRA may reduce the blood glucose level and improve the inflammatory response of patients with DM through regulation of the intestinal flora, mainly Lactobacillus and Clostridium (34). In short, the above studies show that LIRA improves the function of islet β cells in IGT rats, regulates glucose and lipid metabolism, reduces islet cell apoptosis, and modulates the pharmacological effects of intestinal flora through multiple dimensions and pathways.

## 5 Conclusions

In this experiment, TMT quantitative proteomics technology was first used to analyze the biological process of LIRA in treatment of IGT rats. On this basis, 44 potential protein targets, biological processes, and related signaling pathways were identified. In the subsequent experiments, we will continue studying the above-mentioned target proteins and further explore their related mechanisms of action to provide new research ideas and therapeutic directions for the prevention and treatment of IGT.

## Data availability statement

The original contributions presented in the study are included in the article/Supplementary Materials. Further inquiries can be directed to the corresponding authors.

## Ethics statement

The animal study was reviewed and approved by the Animal Ethics Committee of Shandong University of Traditional Chinese Medicine (No. SDUTCM20190520001).

## Author contributions

JL designed the experiments. QG and CH performed the experiments and wrote the manuscript. SZ and YX performed the bioinformatic analyses. QC and XH provided comments. HL supervised the study. All authors contributed to the article and approved the submitted version.

## Funding

This study was supported by the National Natural Science Foundation of China (No: 81673966) and Shandong Province’s Taishan Scholar Project Special Funding (No: ts201712097). Youth Project of TCM Program of Shandong Provincial Administration of TCM in 2022(No. Q-2022051).

## Acknowledgments

We would like to thank all the researchers involved in this study.

## Conflict of interest

The authors declare that the research was conducted in the absence of any commercial or financial relationships that could be construed as a potential conflict of interest.

## Publisher’s note

All claims expressed in this article are solely those of the authors and do not necessarily represent those of their affiliated organizations, or those of the publisher, the editors and the reviewers. Any product that may be evaluated in this article, or claim that may be made by its manufacturer, is not guaranteed or endorsed by the publisher.
